# Likelihood of suffering from an eating disorder in a sample of Spanish cyclists and triathletes

**DOI:** 10.1186/s40337-020-00350-z

**Published:** 2020-11-12

**Authors:** José J. Muros, Ángela Ávila-Alche, Emily Knox, Mikel Zabala

**Affiliations:** 1 Department of Didactics of Corporal Expression, University of Granada, Campus Universitario de la Cartuja s/n, 18071 Granada, Spain; 2 Department of Physical Education and Sport, University of Granada, Granada, Spain; 3 Andalusian School of Public Health (EASP), Granada, Spain

**Keywords:** Elite athletes, Eating disorders, Diet, BMI, Risk factors

## Abstract

**Background:**

During recent years, there has been increasing interest in the study of eating disorders within sports practitioners, with prevalence being reported to be higher than in the general population. The aim of this study was to describe and predict eating disorders according to sex, body mass index, age and sport discipline within a sample of athletes.

**Methods:**

A sample of 4037 cyclists and triathletes from across Spain was selected. Athletes self-reported demographic characteristics and completed the revised restraint scale, SCOFF questionnaire and Mediterranean diet adherence screener. To be eligible for inclusion, participants had to be over eighteen years old.

**Results:**

Males were significantly less likely than females (*p* < 0.001; OR = 0.33), and triathletes (*p* < 0.01; OR = 0.76) were less likely than cyclists to suffer from an eating disorder. Possibility of suffering from an eating disorder increased with increasing body mass index (*p* < 0.001; OR = 1.38) and decreasing age (*p* < 0.001; OR = 0.97).

**Conclusion:**

Findings suggest that the roles of sex, sport discipline, age and body mass index predict risk factors for eating disorders in a sample of Spanish athletes. Clinical diagnosis seems necessary to better understand the factors and mechanisms at play when Spanish athletes develop an eating disorder.

**Trial registration:**

Ethics Committee of the University of Granada (N°883) data: 16/11/2015.

## Plain English summary

Interest has recently increased in looking at eating disorders in people who participate in sport as they may suffer such disorders more than those who do not participate in sport. The aim of this study was to describe eating disorders in a group of athletes. It also looked at whether aspects such as sex, body mass (the ratio between weight and height), age and sport discipline made a disorder more likely.

A total of 4037 cyclists and triathletes from across Spain were selected. Athletes reported demographic characteristics (e.g. age, gender) and completed different questionnaires which measured their eating habits. All participants were over eighteen years old.

Males were less likely than females to have an eating disorder. Also, triathletes were less likely than cyclists to have an eating disorder. Younger individuals and those with a higher body mass index were more likely to have an eating disorder.

In this group of Spanish athletes, it appears that sex, the type of sport an individual participates in, age and body mass influence the chance of having an eating disorder. Clinical diagnosis seems necessary to better understand the reasons why Spanish athletes develop an eating disorder.

## Background

Eating disorders (ED; anorexia nervosa, bulimia nervosa, binge eating disorders and other specified feeding or eating disorders) are a serious mental illness. Anorexia nervosa and bulimia nervosa affect 1–4% and 1–2% of individuals, whilst binge eating disorders are suffered by 1–4% of women [1] and 0.3–0.7% of men in Europe [2]. Worldwide rates are increasing in the general population [3], particularly amongst young people (15 to 19 years old) [4] and eating disorders are associated with some of the highest mortality rates found for mental illnesses [5].

During recent years, there has been an increasing interest in the study of ED within sports practitioners with prevalence being reported to be higher than in the general population [6–8], reaching 45% in female athletes [9]. ED in sport practitioners is also particularly interesting in young people where excessive exercise is used to control calorie intake [10]. Indeed, excessive exercise is a prominent characteristic of many patients suffering from ED [11]. Many adult athletes diagnosed with ED report having started dieting and having developed ED during puberty or adolescence [12]. As a potentially protective factor, other dieting habits such as following Mediterranean dietary patterns have been shown to be inversely associated with risk of anorexia nervosa and bulimia nervosa [13].

This is an especially key period for females who experience a particularly rapid change in body composition [14]. Further, ED developed at this stage are also likely to continue into adulthood [15]. Because of this, athletes are at risk of suffering from a phenomenon known as athlete triad. Female athlete triad concerns the relationship between energy deficiency, menstrual irregularities and low bone mass, however, this is not just a female concern. In males, energy deficits may lead to a reduced testosterone level and low bone mass [9].

Diagnosis of ED in athletes may be underdiagnosed since they often present a subclinical form [16]. Sport practitioners are particularly at risk of suffering ED due to greater sociocultural pressures to conform to body shape ideals. This can also be compounded by pressures from within their specific sport to improve performance [17]. This can be seen through evidence that practitioners competing in sports that encourage leanness seem to present more ED [18] and greater body shape concerns [19] than those involved in sports that do not encourage leanness. ED also vary depending on the type of sport involved in, with greater prevalence being seen in sports with weight classes, aesthetic sports and sports where having a low body mass is sees as advantageous, for example in cycling [8]. Although, cycling ant triathlon are both endurance sports, cycling is more tactical than triathlon. The physical and psychological differences between these sports are not well establish. However, triathletes seem to have higher mental ability and greater motivation to persist in the sport [20].

ED resulting from body image dissatisfaction are typically considered to be a female problem but the focus, nowadays, is evolving to include males [21]. Whilst females are more commonly affected than males [4, 9], in sport, both sexes have a high risk of ED due to the fact that leanness gives a competitive advantage [8]. Specifically, 10% of males participating in endurance sports reported ED [22]. A recent meta-analysis showed a higher incidence of ED in male athletes compared with non-athletes, althoughsome sports seem to present a higher risk than others [23].

ED can be considered a public health problem that affects not only the population in general but also athletes in particular. The aim of this study was, therefore, to describe and predict eating disorders according to sex, body mass index (BMI), age, adherence to MD and sport discipline (cyclists vs. triathlon) within a sample of athletes from Spain.

## Method

### Participants and procedure

This study was conducted during 2016 with a sample of 4037 (36.14 ± 9.28 years) cyclists and triathletes (male: 90.1%) from across Spain. There were 75,871 (male: 95%) federated cyclists in Spain during 2016, of which 2037 (male: 95.5%) satisfactorily completed all questionnaires. In addition, 27,760 (male: 82.3%) triathletes were federated in Spain during 2016, of which 2000 (male: 84.5%) satisfactorily completed all questionnaires. Participants self-reported their demographic characteristics and completed the revised restraint scale (RRS), SCOFF questionnaire (SCOFF; sick, control, one stone, fat and food) (SCOFF) and Mediterranean diet adherence screener (MEDAS) via the Google Drive® (Alphabet, Mountain View, USA) application. The final questionnaire and instructions for completion were sent by e-mail to the Royal Spanish Cycling Federation and to the Spanish Triathlon Federation, who then forwarded to all associated members. To be eligible for inclusion, participants had to be over eighteen years old and have previously given permission to their relevant federation to contact them via email.

Ethical principles of the Declaration of Helsinki for medical research were adhered to. Ethical approval was granted by the Ethics Committee of the University of Granada (N°883).

### Measures

#### Demographics

Participants self-reported their sex, date of birth, competitive level, marital status, height, weight, sport discipline (cyclist, triathlon) and region. Body mass index (BMI) was calculated as weight divided by height squared (kg/m^2^).

### Dietary restraint

The revised restraint scale (RRS) is a 10-item self-reported scale [24] that consists of two subscales assessing dietary restraint: Concern for dieting (CD) and weight fluctuation (WF). Response options are provided on a 4-point Likert scale for the CD subscale, and a 5-point Likert scale for the WF subscale. Scores provide a measure of chronic food restriction and range between 0 and 35. Higher scores indicate higher levels of dietary restraint. In this study, the Spanish version of the RRS [25] was used, which has shown adequate levels of internal consistency for each subscale (CD = 0.68–0.78; WF: 0.71–0.79), with a Cronbach’s alpha of 0.81.

### Eating disorders

The five-item SCOFF questionnaire was used to assess disordered eating behaviour. This is a widely used screening tool [26]. We applied the Spanish adaptation of this questionnaire [27]. The five questions are binary coded (yes/no) with scores ranging from 0 to 5 and ≥ 2 being considered as a positive indication of an eating disorder. Prior studies have determined this cut-off to be both sensitive (72–100%) and specific (73–94%) for the diagnosis of anorexia and bulimia nervosa [26, 28]. Recent research found SCOFF to have 70% sensitivity and 78% specificity for detecting binge EDs [29]. The Spanish version of SCOOF has been found to have 97.7% sensitivity and 94.4% specificity for detecting EDs in primary care [27].

### Mediterranean diet adherence

The Mediterranean diet (MD) adherence screener (MEDAS) [30] was used to determined level of adherence to the MD. Comparative validity of MEDAS compared with the food frequency questionnaire was r = 0.52 and ICC = 0.51. The questionnaire consists of 14 items related to Mediterranean dietary patterns, twelve questions on food consumption frequency and two questions on food intake habits. Each question is scored as 0 or 1, producing a derived score that ranges from 0 to 14. Higher scores indicated greater adherence to the MD. Although MEDAS was designed to evaluate elderly people, it is also suitable for assessing MD adherence in younger adults and adults in general [31].

### Statistical analysis

Means for all quantitative variables are presented alongside standard deviations. Normality of the data was tested using the Kolmogorov-Smirnov test with Lilliefors correction and homoscedasticity was assessed using the Levene test. After verifying that variables were not normally distributed, data were analysed using U Mann-Whitney for two-group comparison. Non-parametric variables are presented according to frequency distributions and associations between them were determined using the Chi-squared test.

Two binary logistic regression models were developed to predict the probability of athletes suffering from an ED and identify restraint scale according to sex, BMI, sporting discipline, age and MD adherence. Model 1 predicted likelihood of suffering from an ED (positive screen and negative screen) with this being entered as the predictor variable. It represented a basic model adjusted for sex, BMI, sporting discipline, age and MD adherence. Model 2 predicted RRS, with this being entered as the predictor variable. It was a basic model adjusted for age, sex, BMI, MD adherence, competitive level and sporting discipline. Model fit was assessed using Pearson chi-squared and Hosmer-Lemeshow tests. Both models demonstrated good fit to data: Model 1: The model X^2^ = 13.44; *p* = 0.10. Model 2: F = 112.15; *p* < 0.001. Data were analysed using IBM-SPSS version 25.0 statistical programme for Windows (Armonk, NY: IBM Corp). The level of significance was set at 0.05.

## Results

### Descriptive statistics

Data for age, BMI, MEDAS score, overall RRS score, RRS subscale scores for CD and WF, and SCOFF scores for all study participants according to sport discipline and sex are shown in Table 1. There were no significant differences according to overall RRS scores and WF subscale scores according to sport discipline. Cyclists were significantly older (P< .001), with higher BMI (P< .001) and SCOFF scores (P< .001), and lower MEDAS (P< .001) and CD scores (P< .01) than triathletes. Males reported being older (P< .001) and had higher BMI (P< .001) and WF scores (P< .001), and lower MEDAS (P< .001), CD (P< .001) and SCOFF scores (P< .001) than females. No significant differences were reported regarding RRS scores.

**Table 1:** Characteristics of the study sample

	Cyclists (***N*** = 2037)	Triathletes (***N*** = 2000)	***p*** value	Male (***N*** = 3634)	Female (***N*** = 401)	***p*** value
**Age (years ± SD)**	37.72 ± 9.67	34.54 ± 8.58	<.001	36.60 ± 9.15	31.96 ± 9.40	<.001
**BMI (kg/m**^**2**^ **± SD)**	23.74 ± 2.69	22.85 ± 2.28	<.001	23.54 ± 2.46	21.08 ± 2.16	<.001
**MEDAS (points ± SD)**	7.44 ± 2.12	7.85 ± 2.10	<.001	7.59 ± 2.11	8.15 ± 1.99	<.001
**Overall RRS (points ± SD)**	21.97 ± 5.34	22.14 ± 5.21	.269	22.02 ± 5.25	22.41 ± 5.53	.319
**CD (points ± SD)**	12.15 ± 3.19	12.41 ± 3.22	.008	12.12 ± 3.11	13.68 ± 3.71	<.001
**WF (points ± SD)**	9.83 ± 3.28	9.73 ± 3.20	.343	9.90 ± 3.25	8.74 ± 2.93	<.001
**SCOFF (points ± SD)**	.74 ± 1.09	.62 ± .99	<.001	.66 ± 1.03	.87 ± 1.14	<.001
**Experience (years)**	12.98 ± 10.03	5.56 ± 6.31	<.001	9.66 ± 9.31	6.13 ± 7.17	<.001
**Training (hours per week)**	10.94 ± 4.64	11.71 ± 4.84	<.001	11.34 ± 4.71	11.14 ± 5.12	.278

*BMI* Body mass index, *MEDAS* Mediterranean diet adherence screener, *RRS* Revised restriction scale, *CD* Concern for dieting, *WF* Weight fluctuation, *SCOFF* Five-item SCOFF questionnaire, *SD* Standard deviation

Table 2 shows the proportion of athletes at risk of suffering an ED according to sex and discipline. Females and cyclists showed a greater likelihood of suffering from an ED than males (23.2% vs. 17.1%) and triathletes (19.8% vs. 15.6%).

**Table 2:** Proportion of athletes at risk of eating disorders according to sex and discipline

	SCOFF < 2 points	SCOFF ≥ 2 points	***P*** value
**Sex**			
Male (3636)	3015 (82.92%)	621 (17.08%)	
Female (401)	308 (76.81%)	93 (23.19%)	.002
**Discipline**			
Triathlon (2000)	1689 (84.45%)	311 (15.55%)	
Cycling (2037)	1634 (80.22%)	403 (19.78%)	<.001

### Prediction of suffering from an eating disorder

A binary logistic regression model was constructed to predict participants likelihood of suffering from an ED. The variable describing whether participants suffered from an ED formed the dependent variable. Sex, BMI, sporting discipline, age and Mediterranean diet adherence provided the independent variables. Competitive level, living status and region were excluded as they did not contribute to the final model (Table 3).

**Table 3:** Binary logistic regression to predict eating disorder risk

Analysis	Variable	b	Standard Error	Wald X^2^	Exp(B)	df	p	CI
SCOFF risk	Sex	−1.09	0.14	57.98	0.34	1	0.00	0.25–0.44
	Discipline	−.25	0.09	7.59	0.78	1	0.01	0.65–0.93
	Age	−.04	0.01	45.07	0.97	1	0.00	0.96–0.98
	MD adherence	0.03	0.02	1.70	1.03	1	0.19	0.97–1.07
	BMI	0.32	0.02	266.52	1.38	1	0.00	1.33–1.43

*MD* Mediterranean diet, *BMI* Body mass index

The model indicates that all of the included dependent variables significantly predicted ED likelihood apart from Mediterranean diet adherence. Males were significantly less likely than females (*p* < 0.001; OR = 0.33) and triathletes (*p* < 0.01; OR = 0.76) were less likely than cyclists to suffer from an ED. Likelihood increased with increasing BMI (*p* < 0.001; OR = 1.38) and decreasing age (*p* < 0.001; OR = 0.97). The model was found to explain 13.1% of the variance in the dependent variable. The model demonstrated good fit (X^2^ = 13.44; F = 109.98; *p* = 0.10).

### Prediction of likelihood of reporting a high restraint scale score

A linear regression model was constructed to predict participating athletes’ likelihood of reporting high RRS values. In this case the dependent variable was RRS evaluation score with the same independent variables being considered as in model 1. Age, sex, BMI, MD adherence, competitive level and sporting discipline provided the independent variables. Living status and region were again excluded as they did not contribute to the model (Table 4).

**Table 4:** Linear regression model to predict risk of reporting high restraint scale

Analysis	Variable	B	Standard Error	t	Beta	p	CI
RS score	Age	−0.049	0.01	−5.21	−0.09	< 0.01	−0.07 – −0.03
	BMI	0.86	0.03	25.45	0.41	< 0.01	0.79–0.93
	Sex	−2.00	0.27	−7.31	−0.11	< 0.01	−2.53 - -1.46
	MD adherence	0.23	0.04	6.01	0.09	< 0.01	0.15–0.30
	Category	0.21	0.24	0.88	0.01	0.38	−0.26 – 0.68
	Discipline	0.46	0.16	2.86	0.04	< 0.01	0.14–0.77

*MD* Mediterranean diet, *BMI* Body mass index

The model indicated that all variables significantly predicted RRS score apart from competitive level. Participating athletes who were younger (β = − 0.09; *p* < 0.001), had a higher BMI (β = 0.41; *p* < 0.001) and had higher MD adherence (β = 0.09; *p* < 0.001) reported higher RRS scores. Males reported lower RRS scores than females (β = − 0.11; *p* < 0.001) and triathletes reported higher scores than cyclists (β = 0.04; *p* < 0.01). The model explained 14.3% of the variance found in the dependent variable and demonstrated good fit to the observed data (X^2^ = 16.06; F = 112.15; *p* < 0.001).

## Discussion

The present study highlighted a large proportion of athletes who screened positive for an ED, finding such disorders to be related to sex, sport discipline, age and BMI.

In this national sample of Spanish athletes (cyclists and triathletes), 17.9% were shown to be have a higher likelihood of suffering from an ED, with females and cyclists presenting higher prevalence when compared with males and triathletes. To our knowledge, this is the first study suggesting that sport discipline (cyclists and triathletes) might have an influence on the detection of ED likelihood, independent of sex, BMI and age.

A recent study involving collegiate male athletes from the United States showed that basketball, cycling and wrestling emerged as sports with the highest proportion of players reporting clinically elevated ED examination-questionnaire scores [32]. Seventeen percent of cyclists reported global ED scores within a clinical range. The prevalence rate in their study is similar to that seen in the present study for male athletes (17.08%). In the only other study to evaluate cyclists, male cyclists scored higher than male non-cyclists. In this study, 19.7% of male cyclists met or exceeded a score of 20 on the eating attitude test-26 (EAR-26) [33]. This being slightly higher than in the present study. With regards to triathletes, the only other study to evaluate sub-clinical ED did so in a sample of 583 male and female triathletes. This study found that 28% of evaluated females and 11% of evaluated males scored below the mid-point of the range on the EAT-26 [34]. Prevalence in the present study was slightly lower for females and higher for males. The reason for this discrepancy could be related with the tool used to evaluate ED or be a result of the comparatively higher sample size included in the present study.

The present study showed a higher likelihood of ED in cyclists than triathletes. This may be explained by the watt/kilo ratio, which has a special impact on climbing performance in cycling. However, this aspect is also important for triathletes in the running race. Thus, the reason could, in fact, be more related with cultural or social aspects rather than the nature of the sport itself. We therefore urge researchers to consider the specific characteristics of their sample, rather than categorise individuals within global sports using simplistic categorization criteria such as ‘endurance sport’.

The present study shows a higher likelihood of ED amongst females. Other studies have found similar results. Pustivšek, Hadžić, Dervišević & Carruthers (2019) [35] also observed that female cyclists and triathletes were significantly more likely than males to have a high ED prevalence. Similar results have been found in a sample of adolescent female and male athletes. In this case, the proportion was 1:3.5 amongst distance runners, with both males and females being at risk of ED, though with females exhibiting higher risk [36]. Similar results were found by Sundgot-Borgen & Torstveit (2004) [8] who reported that 20% of female athletes and 8% of male athletes met criteria for an ED, compared to 9% of female controls and 0.5% of male controls. Different results were found in a study involving 1031 German endurance athletes. In this case, 18.9% of athletes surveyed were at risk of developing an ED, this being slightly higher than in the present study. However, the former did not find gender differences related to the probability of developing an ED [37]. A more recent study did not find any differences in elite male or elite female football players [38]. It seems clear that the female population has a higher risk of suffering from an ED than the male population. Nevertheless, ED appear more frequently in male athletes than in male non-athletes [8]. Controversial results have been shown in samples of sport practitioners. Therefore, it seems important to study the prevalence of ED in male athletes as well as in female as prevalence seems to be high in both in comparison with non-athletes, with this having a devastating effect on athlete health and performance [39].

The present study showed that the likelihood of suffering ED increased with BMI. Weight status (normal weight, underweight, overweight and obese) was also considered but BMI was seen to predict a far greater proportion of variance and weight status did not produce significant results. These results are interesting as we hypothesized that respondents who were underweight or overweight would both be more susceptible to ED but, in this case, only high BMI was significant. A recent study [35] with adolescent Slovenian athletes showed that ED at-risk groups had significantly higher BMI percentiles and fat mass percentages, and lower muscle mass and fat-free mass percentages. Another study involving Spanish adolescents found that overweight adolescents had a higher risk of developing ED than non-overweight adolescents. However, overweight adolescents with high levels of physical fitness had a lower risk of ED than overweight adolescents with low levels of physical fitness [40]. Thus, it seems that anthropometric parameters could be a better way to predict ED risk in athletes than weight status.

With regards to age, the likelihood of suffering from an ED increased with decreasing age (*p* < 0.001; OR = 0.97). To our knowledge this is the first study to analyse ED likelihood in a sample of adults. The majority of researchers focus their research on the adolescent stage at this is seen to be a period during which individuals have a higher possibility of suffering from an ED. A recent study showed that the percentage of Spanish adolescents with a SCOFF+ score indicating presence of an ED was 21.7% (28.1% in girls and 11.2% in boys) [41]. This result is similar to those produced by another study of adolescents from Spain (22.8% in 2007 [42].

The main limitation of the present study is its cross-sectional design as this cannot establish casual relationships. The results must be interpreted with caution because we only had self-reported measurements. Although the sample was large, self-selection bias means that generalizability is limited. Further, the present study only assessed prevalence of ED via self-report questionnaire. Although SCOFF is a widely used screening tool with good sensitivity and specificity, it can only discern individuals at greater likelihood of developing ED, complete diagnosis requires clinical follow-up. Thus, future research is needed to determine whether those who engage in endurance sport (cyclists and triathletes) are more likely to be diagnosed with clinical ED and whether prevalence is greater amongst cyclists, female athletes and those with a high BMI. Weight and height were self-reported as opposed to directly measured due to time, financial resource and manpower constraints. While this method will be less accurate than direct measurement it has demonstrated good agreement and validity in healthy weight populations. Whilst BMI may not always be a reliable measure for athletes due to its failure to differentiate between lean body mass and fat mass, literature on endurance athletes, such cyclists and triathletes have shown that BMI is comparable with the normal population. BMI is, therefore, an appropriate instrument for detecting health-related weight problems. Besides, BMI is easy to calculate, predictive of obesity-related diseases and is the recommended screening tool for overweight and obesity in large populations.

## Conclusions

Results of the present study show a lower prevalence than that within adolescents but still indicates a high prevalence. Although adolescence is a critical period for ED, we must pay attention to adult athletes as well, placing a special focus on young adults.

The present study provides evidence for the role of sex, sport discipline, age and BMI as predictors of ED likelihood in a sample of Spanish athletes (cyclists and triathletes). Clinical diagnosis seems necessary to better understand the risk factors at play when Spanish athletes develop ED.

## Data Availability

The datasets used and/or analysed during the current study are available from the corresponding author on reasonable request.

## Abbreviations

BMI: Body mass index
CD: Concern for dieting
ED: Eating disorders
MD: Mediterranean diet
MEDAS: Mediterranean diet adherence screener
RRS: Revised restraint scale
SCOFF: Sick, control, one stone, aft and food
WF: Weight fluctuation
